# Distinct Molecular Signature of Bovine Spongiform Encephalopathy Prion in Pigs

**DOI:** 10.3201/eid1601.091104

**Published:** 2010-01

**Authors:** Torsten Seuberlich, Andreas Zurbriggen

**Affiliations:** University of Berne, Berne, Switzerland

**Keywords:** Bovine spongiform encephalopathy, BSE, cattle, prion, prion protein, pig, TSE, prions and related diseases, scrapie, letter

**To the Editor:** In a recent article in Emerging Infectious Diseases, Espinosa et al. (*1*) investigated the porcine transmission barrier to infection with bovine and ovine transmissible spongiform encephalopathies (TSEs) in transgenic mice expressing the porcine prion protein. Bovine spongiform encepatholopathy of the classical type (BSE) derived from cattle and sheep, as well as atypical scrapie, transmitted to these mice, although with different efficiencies. Whereas sheep BSE showed a 100% attack rate, cattle BSE and atypical scrapie showed a higher transmission barrier in the first passage. Unexpectedly, the electrophoretic profile of the proteinase K–resistant prion protein (PrP^res^) in Western immunoblot (WB) analysis of all 3 TSEs shifted toward a common signature upon transmission. This was a 3-band pattern with a predominant monoglycosylated PrP^res^ moiety and, therefore, clearly differed from those of the BSE and atypical scrapie inocula. The authors speculated that the porcine cellular prion protein (PrP^c^) might allow only for few options as it changes its conformation to the disease-associated prion protein. However, whether this effect is attributable to the porcine PrP^c^ transgene or to the genetic background of the mouse model remains unknown.

To our knowledge, BSE has been successfully transmitted to pigs in 1 study, but WB data were not reported (*2*). We had access to central nervous system tissues of 1 of these animals (kindly provided by the Veterinary Laboratories Agency TSE Archive, Weybridge, UK) and aimed at assessing whether a similar effect occurs when cattle BSE affects pigs. Our results show a PrP^res^ signature in BSE-infected pigs similar to that described for the porcine PrP^c^ transgenic mice and clearly different from that in cattle (Figure). These findings support the finding by Espinosa et al. that the molecular shift most likely was due to intrinsic properties of the porcine PrP^c^. Therefore, in this respect the mouse model appears to reflect the situation in the pig.

**Figure:**
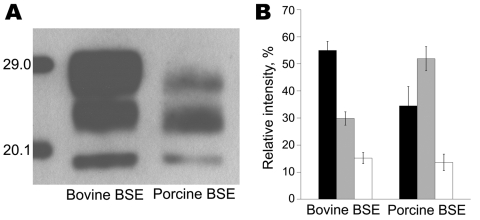
Molecular signature of bovine spongiform encephalopathy (BSE) in pigs. A) Comparative Western immunoblot analysis of the proteinase K–resistant core fragment (PrP^res^) of the pathologic prion protein in BSE in cattle and in an experimentally BSE-infected pig using the monoclonal antibody 6H4 (Prionics, Schlieren, Switzerland). B) Average relative intensities of the diglycosylated (black bars), monoglycosylated (gray bars), and unglycosylated (white bars) PrP^res^ moieties as determined by the Quantity One software package (Bio-Rad, Rheinach, Switzerland). Data are based on 4 independent runs, and error bars indicate SD. Note the different extent of PrP^res^ glycosylation in bovine and porcine BSE. By contrast, the molecular masses of the unglycosylated PrP^res^ were similar and scored 18.89 kDa (SD ± 0.28 kDa) and 18.90 kDa (SD ± 0.42 kDa) in bovine and porcine BSE, respectively. Molecular masses of the standards are indicated on the left in panel A.

BSE prions are considered to transmit to other species, such as exotic ruminants, cats, macaques, humans, sheep, and goats, without any obvious alterations of the molecular phenotype (*3**,**4*). Our study provides evidence that the molecular phenotype of classical BSE also may shift upon genuine interspecies transmission. Attempts to discriminate BSE from other prion diseases in humans and animals often rely at first on the analysis of the PrP^res^ signature in WB. Consequently, the situation described in our study complicates the interpretation of such disease surveillance data to assess public health risks for animal TSEs. Whether this applies to other TSEs and species remains to be addressed.

## References

[1] Espinosa JC, Herva ME, Andreoletti O, Padilla D, Lacroux C, Cassard H, Transgenic mice expressing porcine prion protein resistant to classical scrapie but susceptible to sheep bovine spongiform encephalopathy and atypical scrapie. Emerg Infect Dis. 2009;15:1214–21. 10.3201/eid1508.081218 19751582 PMC2815954

[2] Wells GA, Hawkins SA, Austin AR, Ryder SJ, Done SH, Green RB, Studies of the transmissibility of the agent of bovine spongiform encephalopathy to pigs. J Gen Virol. 2003;84:1021–31. 10.1099/vir.0.18788-0 12655106

[3] Collinge J, Sidle KC, Meads J, Ironside J, Hill AF. Molecular analysis of prion strain variation and the aetiology of ‘new variant’ CJD. Nature. 1996;383:685–90. 10.1038/383685a0 8878476

[4] Hill AF, Desbruslais M, Joiner S, Sidle KC, Gowland I, Collinge J, The same prion strain causes vCJD and BSE. Nature. 1997;389:448–50. 10.1038/38925 9333232

